# Chromatin Insulators and Topological Domains: Adding New Dimensions to 3D Genome Architecture

**DOI:** 10.3390/genes6030790

**Published:** 2015-09-01

**Authors:** Navneet K. Matharu, Sajad H. Ahanger

**Affiliations:** 1 Department of Bioengineering and Therapeutic Sciences, Institute for Human Genetics, University of California San Francisco, San Francisco, CA 94143, USA; 2 Department of Ophthalmology, Lab for Retinal Cell Biology, University of Zurich, Wagistrasse 14, Zurich 8952, Switzerland

**Keywords:** insulators, architectural proteins, TAD boundaries, chromatin architecture

## Abstract

The spatial organization of metazoan genomes has a direct influence on fundamental nuclear processes that include transcription, replication, and DNA repair. It is imperative to understand the mechanisms that shape the 3D organization of the eukaryotic genomes. Chromatin insulators have emerged as one of the central components of the genome organization tool-kit across species. Recent advancements in chromatin conformation capture technologies have provided important insights into the architectural role of insulators in genomic structuring. Insulators are involved in 3D genome organization at multiple spatial scales and are important for dynamic reorganization of chromatin structure during reprogramming and differentiation. In this review, we will discuss the classical view and our renewed understanding of insulators as global genome organizers. We will also discuss the plasticity of chromatin structure and its re-organization during pluripotency and differentiation and in situations of cellular stress.

## 1. Introduction

To fit into the small volume of the nucleus, eukaryotes package their relatively large genome compactly into a nucleo-protein complex called chromatin. This packaging must ensure that specific regions in the genome remain accessible to large protein complexes that are responsible for important cellular processes such as transcription, replication, recombination, and DNA repair. At the same time, the effect of non-coding regulatory elements such as enhancers and repressors needs to be restricted to their cognate genes, preventing inappropriate activation or repression of neighboring expression domains. Additionally, developmental and tissue-specific expression demands dynamic reorganization of chromosomal domains at specific times and in specific subsets of cells. Eukaryotic genomes have evolved to organize chromatin fiber into a series of topologically and functionally independent domains. The structural organization of chromosomes at the interphase nucleus correlates with the transcription status of genomic regions. Highly transcribed regions are less dense and are placed towards the interior of the nucleus. DNA-FISH experiments have shown that different regions within the chromosome territory usually do not “intermingle” and therefore form separate compartments within a single chromosome [1]. The formation of such autonomous compartments in the chromosome can be achieved in a variety of ways, including establishment of a physical block to *cis* spreading of a chromatin state, recruitment of specific activities to a limited locus, or targeting to a sub-nuclear compartment associated with either silencing or activation. The study of factors and processes responsible for the formation and maintenance of such autonomous compartments is an active area of investigation. Specialized regulatory elements, termed “boundaries” or “insulators,” have emerged as likely candidates to play this crucial role of chromatin compartmentalization. Such elements have been characterized by two experimentally defined properties involving altered gene expression. First, an insulator element acts as an enhancer-blocker by disrupting enhancer-promoter interactions, when positioned between an enhancer and a promoter, without rendering the enhancer inactive (as it is still capable of activating a “non-insulated” promoter) [2,3]. Second, when flanking a transgene, insulators are able to protect the transgene from position effects, particularly from the repressive effects of heterochromatin, allowing for position-independent gene expression [4]. This property of insulators is often referred to as barrier activity, since it involves blocking the spread of one chromatin state into another [5]. Some of the characterized boundaries have been shown to act primarily as barriers to heterochromatin in yeast, whereas others may possess both properties, *i.e.*, enhancer-blocking and barrier activity [6]. It is notable that, while barrier activity of boundaries prevents transcriptional repression, their enhancer-blocking property interferes with transcriptional activation. The two properties that define boundaries originate from the experimental assays used to identify and characterize them.

Insulator sequences and their associated proteins have been identified across species ranging from yeast to mammals. In yeast, TFIIIC is well characterized for its role in preventing spreading of repressive chromatin at RNA PolIII transcribed tRNA genes [7]. TFIIIC associates with some of the insulator elements in higher eukaryotes also. In mammals, a zinc-finger protein, CTCF, binds to most of the known insulator sequences. Several studies have shown that CTCF is involved in mediating chromatin interactions at several genomic loci in mice and humans that include but are not limited to beta-globin, H19/Igf2, MHC-II, HoxA, *etc*. [8]. It has also been found that both TFIIIC and CTCF require Cohesin for their insulator function in yeast and mammals, respectively. Studies have shown that tRNA genes can also function as enhancer-blockers in mammals, and that CTCF co-localizes with TFIIIC at several genomic loci, indicating a conserved mechanism of insulator function [9,10,11]. While many components of the insulator tool-kit are similar, there is considerable species-specific compositional variation. This is best reflected in *Drosophila*, which has evolved a diverse set of insulator factors and co-factors. These include the *Drosophila* homologue of vertebrate CTCF (dCTCF), Zest-white-5 (Zw5), Boundary Element Associated Factor-32 (BEAF-32), Suppressor of Hairy-wing [Su(Hw)], GAGA factor (GAF) and the recently identified proteins, Pita, and the zinc-finger protein interacting with CP190 (ZIPIC) [12,13,14]. These insulator binding proteins recruit co-factors such as Centrosomal Protein-190 (CP190) and Mod(mdg4), which are critical for their function [15,16]. This complexity of insulator tool-kit is well investigated in *Drosophila*; however, in mammals only CTCF has been studied as an insulator factor. It is possible that we are still far from our understanding of complex insulator functions in mammals. Recently, vertebrate GAF homologue has been investigated to function as an insulator component in mouse cells [17,18]. It has been also shown to tether chromatin loops to the nuclear periphery along with histone de-acetylase complex to repress gene expression [19]. Future studies are expected to enhance our understanding of genome insulation mechanisms in higher vertebrates.

## 2. Chromatin Insulators as a Genome Architectural Tool-Kit

The most widely accepted model of insulator function proposes that boundaries establish physical organization of the chromatin fiber into independent structural and functional domains. This model is based on the assumption that boundaries interact with each other or with components of “chromatin anchor points” such as nuclear matrix or nuclear envelope/lamina to loop out chromatin, providing steric and topological hindrance to enhancer-promoter interaction. The gypsy insulator in *Drosophila* provided one of the first illustrative examples of this model. A mutation in the *mod(mdg4)* gene that encodes one of the components of the gypsy insulator complex acts as an enhancer of position effect variegation (PEV) and has properties characteristic of the trithorax-group (trx-G) genes [20,21]. Additional support is presented by the fact that both Su(Hw) and Mod(mdg4) are found to be distributed at hundreds of sites on polytene chromosomes in salivary glands [22]. Given their distribution across the chromosome, one would expect to see a diffuse homogenous pattern of boundaries in the interphase chromosome of a diploid cell. Interestingly, this is not the case; instead, gypsy insulator proteins accumulate at a small number of nuclear locations. This observation has led to a suggestion that each of these sites where Su(Hw) and Mod(mdg4) proteins accumulate in the nucleus is actually several individual sites coming together, probably through their interaction with each other and/or with the components of other nuclear structure like lamina. However, the aggregation of these sites is not random; in fact 75% of them are present adjacent to nuclear lamina, suggesting that formation of such aggregates may require interaction between gypsy protein components and the nuclear lamina [23]. The tethering of gypsy insulators at the nuclear periphery, suggests that gypsy insulators might be component of MARs/SARs [22]. This idea is supported by the finding that sequences containing the gypsy insulators possess MARs activity [24]. This arrangement of gypsy insulators might create physically and topologically independent domains that interfere with the transmission of the signal from an enhancer located in one domain to a promoter located in the adjacent domain. Other insulator proteins in *Drosophila* such as BEAF 32B (Boundary element associated factor, isoform 32B) binds to scs and scs’ boundary elements and tether these regions to nuclear lamina [25]. Loss of BEAF leads to abrogation of the boundary activity of scs and scs’ regions and hence dissociation from nuclear lamina. Although boundaries primarily organize chromatin by physically restricting the legitimate enhancer-promoter communication, it is to be noted that not all insulators work via tethering chromatin to nuclear matrix or lamina proteins to bring about this organization.

All known vertebrate boundaries require CTCF for their insulator function. CTCF has been shown to target boundaries of the β-globin locus in chicken, mice, and humans to nucleolar periphery [26,27]. Co-purification studies have shown that CTCF co-purifies with nucleolar proteins present at the nuclear periphery. It was shown that CTCF interacts with nucleophosmin in nuclear matrix [26]. Nucleophosmin localizes at CHS4 insulators *in vivo*, along with other CTCF binding sites in the genome [26]. In addition, many CTCF-dependent boundary elements are known to tether to nuclear periphery. It was proposed that, like in *Drosophila*, CTCF-associated insulator complex tethers chromatin to nuclear matrix and thus subdivides chromosomes into active and inactive looped domains in mammals. Using 3C (chromosome conformation capture) assay, long-range interactions were shown to occur between murine β-globin locus and enhancer elements in the LCR, 40 to 60 kb away [28]. This particular interaction was only seen in erythroid cells, where β-globin locus was transcriptionally active [29,30]. At an earlier developmental time point in mice, the same LCR shifts its interaction with other globin genes, switching its transcriptional program in a developmental stage-specific manner [29]. Similar results were observed using RNA-TRAP to demonstrate that the active β-globin locus and enhancers of the LCR come in close contact *in vivo* [31]. The interaction of LCR with globin gene promoter is mediated by CTCF [30]. However, there is no direct evidence that this interaction involves nuclear matrix components.

CTCF-dependent chromatin boundaries of the imprinted Igf2/H19 locus have also been shown to function through the formation of chromatin loops. The parent-specific expression of Igf2/H19 was shown to be mediated through the establishment of chromatin loop domains formed by specific interactions between two differentially methylated regions (DMR) of H19 and Igf2 loci [32]. The differential interaction of H19 DMR and Igf2 DMRs generates a simple epigenetic switch for Igf2 transcription, through which it is localized to either inactive or active nuclear compartments. While in active state, the Igf2 gene promoter lies close to the downstream enhancers of the H19 locus, but not in its inactive state. These results suggest that boundaries may have an ability to relocate specific domains to the new nuclear environment as part of their mechanism of action.

It is emerging from the above studies that insulator elements function by organizing chromatin fiber into independent domains of gene activity. However, some results do not fit well with the structural model for insulation. For example, as with the gypsy insulator elements, interaction of the scs and scs’ boundaries could explain their insulator function; however, the interaction between scs and scs’ also depends upon the adjacent sequences, which may have insulation properties [33]. For example, homologous pairing of gypsy insulators or heterologous pairing of gypsy and binding sites for GAGA factor have been shown to neutralize insulator activity [34]. When 11 homologous and heterologous combination of boundaries were tested, it was seen that heterologous combination of gypsy with other boundaries or homologous pairing of other insulator elements such as scs or SF1 do not always reduce their enhancer-blocking activity [35]. In fact, some paired insulator elements exhibit increased enhancer-blocking activity, suggesting that they can function additively or independently. These early observations developed the “promoter-decoy model” for insulation. According to this model, an insulator sequence is essentially capable of recruiting the components of transcription machinery and hence can compete with the promoter to interact with the enhancer [36]. Co-localization of looped H19ICR (H19 Imprinting control region) insulator with its promoter and the trapping of HS2 enhancer complex of the human ε-globin gene by the inserted insulator substantiate the proposition of a promoter decoy model [37,38]. In the case of ε-globin gene, the HS2 enhancer delivers the transcription machinery to its promoter by tracking along intervening DNA sequence. An insulator inserted between the HS2 enhancer and the globin gene promoter stalls the transcription machinery at insulator site, thereby blocking long-range enhancer function [38]. Nevertheless, not all observed promoter-insulator interactions are explained by the promoter decoy model alone and there is no single inclusive model of insulator function [37]. Recently, the chromatin loop domain model has gained attention owing to its technological advancements, and hence it remains the most plausible model to explain the majority of enhancer-promoter interactions.

## 3. Insulators Organize Topological Domains of the Genome

**Figure 1:**
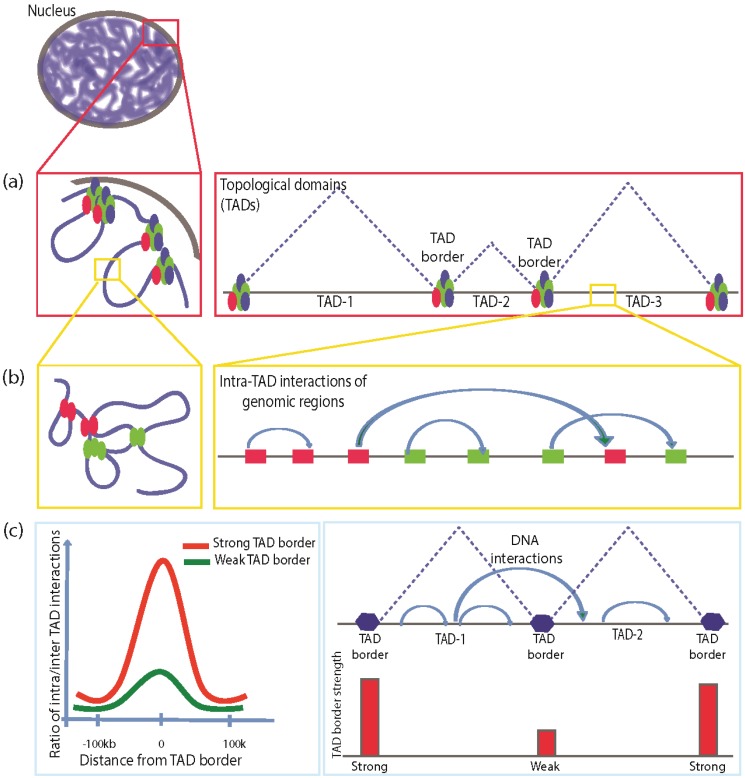
Chromatin nuclear organization with perspective of TAD structuring. (**a**) Megabase-scale chromatin looping and associated topological domain structuring. Insulator factors (shown in colored ovals) are decorated over chromatin mediating the Mb level chromatin looping; (**b**) Kilobase-scale chromatin looping mediated by insulator factors and associated co-factors facilitates specific interactions of gene regulatory regions. Kb-scale looping defines the transcriptional network on the genome; (**c**) TAD border strength and the DNA interaction frequency. A stronger TAD border is defined by the lesser frequency of inter-TAD interactions.

Advances in genome technologies such as ChIP-seq, Chromosome Conformation assays (4C, 5C, Hi-C), and ChIA-PET have rapidly enhanced our understanding of the 3D architecture of the genome [39,40,41,42,43,44,45]. Chromatin conformation capture studies have revealed that the genomes of both vertebrates and invertebrates are divided into megabase to sub-megabase size chromatin domains (Figure 1a), within which smaller sub-domains are formed by local interactions at kilobase scale (Figure 1b). The large Mb scale domains are also termed “Topologically associated domains” or TADs. In *Drosophila* TADs are smaller than those of vertebrates, ranging from tens of kilobases to less than a megabase in structure [46]. They are largely conserved not only in multiple cell types but also across different species and are thought to serve an architectural role in the nucleus. It has been observed that the frequency of intra-TAD interactions is higher, while inter-TAD interactions are generally low. This has been attributed to flanking regions of the TADs termed as TAD-borders, which have low to very low interaction frequencies. Depending on the scale of DNA interaction frequency, these TAD borders can be strong or weak (Figure 1c). The TAD border strength is calculated as the ratio of intra/inter TAD interactions [47]. Data obtained using Hi-C have shown that there is a negative correlation between the strength of the TAD boundary and the frequency of the inter-TAD interaction. A stronger TAD boundary ensures lesser interaction frequency between neighboring TADs. Some of the TAD borders or “TAD boundaries” are rich in tRNA genes, SINE elements (both known to possess insulator activity), and housekeeping genes [48]. They also harbor DNase hypersensitive sites and have high occupancy of insulator proteins [47].

In *Drosophila*, several proteins such as BEAF-32, CTCF, Su(Hw), DREF, Rad21 (cohesin), TFIIIC, Cap-H2 (condesin II), Mod(mdg4), CP190, Z4, Chromator, L(3)mbt, and Fs1h-L were investigated for their binding on TAD borders [47]. While CTCF is known to mediate long-range enhancer-promoter interactions as well as acting as RNA PolII stalling site [49], it was also found to frequently associated with TFIIIC, Cohesin, and PRDM5 at TAD boundaries. Barring few sites, Su(Hw), on the other hand, was generally absent from TAD borders and mostly localized within TADs. Interestingly, strong TAD borders correlated with high occupancy of insulator factors. TAD borders that have high occupancy of insulator factors showed robust enhancer-blocking activity, while those with fewer insulator-binding sites behaved as weak enhancer-blockers [47]. These results suggest that genomic regions that have a high concentration of bound insulator factors (such as TAD boundaries) may serve genome architectural roles. However, we still do not know if TAD borders associate with nuclear lamina to loop out chromatin, as discussed in the previous section.

We are still in the early stages of our understanding of the composition of mammalian TAD boundaries. Only a fraction of CTCF sites are present at the mammalian TAD boundaries, while majority of the CTCF sites are found within TADs [48]. Several studies have shown that CTCF and Cohesin can differentially modulate inter and intra-TAD interactions. For example, loss of Cohesin in HEK289T and mouse thymocytes was shown to result in loss of intra-TAD interactions without affecting TAD architecture [50,51]. However, depletion of CTCF not only affected intra-TAD interactions but also led to loss of TAD boundaries and resulted in an increase in inter-TAD interaction [50]. Other studies have shown that depletion of Cohesin resulted in overall loss of both inter- as well as intra-TAD interactions by affecting Cohesin/CTCF-anchored sites [52]. CTCF and Cohesin are also involved in mediating enhancer-promoter looping; therefore, the loss of either or both results in the abrogation of enhancer-promoter interactions [53]. Future studies are expected to shed more light on the components of TAD structuring in mammals. Taken together, these results suggest that eukaryotic genome is organized by twofold architectural looping; (1) at the Mb-scale, it is organized into topological domains (TADs); and (2) each TAD is further organized at the Kb-scale, to facilitate more local interactions between gene regulatory elements necessary for generating unique transcriptional outputs [54,55]. The role of insulator factors at both levels of genome organization is beginning to emerge. Insulator factors binding to TAD borders at high concentrations may act as “super-insulators” to maintain Mb-level TAD structures. However, at the kilobase scale, insulator factors along with other transcription factors may facilitate more local looping interactions within the TAD structures. Therefore, at least in *Drosophila*, most of the typical “insulator factors” are emerging to be “genome architectural proteins”, thus deviating from their classical definition.

**Figure 2:**
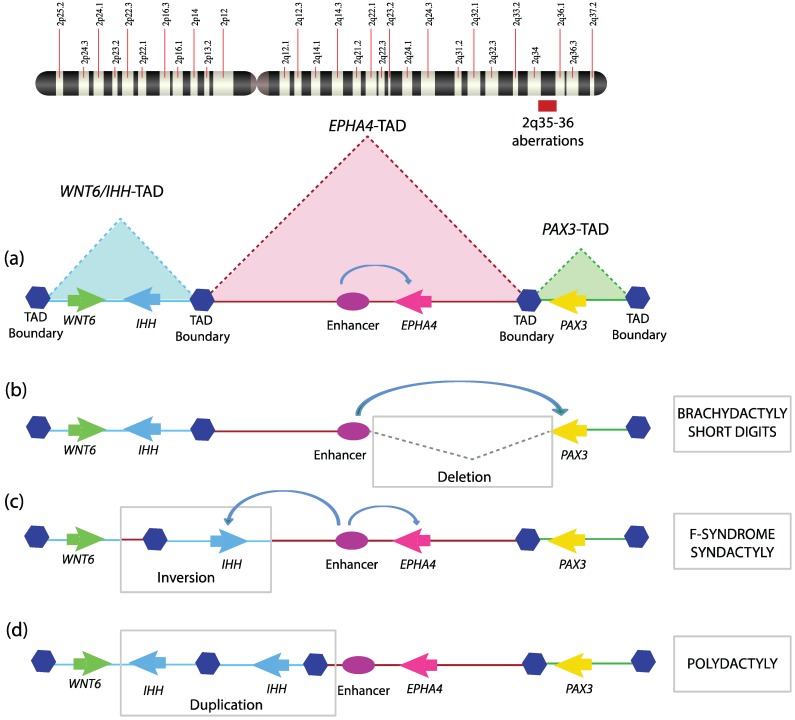
Topological domain disruption linked to limb malformations. Mutations encompassing the *WNT6-IHH/EPHA4/PAX3* locus on human chromosome 2q arm disturb TAD structuring and are thus involved in limb phenotypes; (**a**) The *WNT6-IHH/EPHA4/PAX3* locus is organized into three TADs with intra-TAD specific interactions playing a key role in setting up the transcriptional program during limb development; (**b**) A deletion encompassing the TAD boundary between *EPHA4/PAX3* leads to mis-assignment of *EPHA4* enhancer to *PAX3* promoter and hence short digits; (**c**) Inversion surrounding *IHH* and its nearest TAD boundary allows *EPHA4* enhancer to drive the IHH promoter, which was prevented by structural hindrance posed by the TAD boundary in a wild-type scenario, hence leading to F-syndrome; (**d**) Duplication of *IHH*/TAD boundary results in extra TAD, which causes the polydactyly phenotype.

Given the critical role of TAD boundaries in genome organization, it is not surprising that genomic defects that disrupt TAD boundaries can cause debilitating diseases. A number of human diseases are associated with chromosomal aberrations such as deletion, inversion, translocation, and duplication. To what extent such genomic variations affect chromatin topology and their subsequent contribution to the disease phenotype is nearly untouched. A recent study demonstrated how structural anomalies in the genome could disrupt TAD organization and result in at least three related human genetic disorders [56]. Three different types of limb malformations, namely brachydactyly (short digits), F-syndrome syndactyly (fused axial digits), and polysyndactyly (duplicated and fused digits), identified in three different families, were investigated. By performing comparative genomic hybridization (CGH), the above mentioned malformations were shown to be associated with genomic aberrations in the q arm of chromosome 2, having four important coding genes, WNT6, IHH, EPHA4, and PAX3. Investigating the TAD organization of the locus revealed that it is structurally divided into three independent TADs, PAX3-TAD, EPHA4-TAD, and WNT6/IHH-TAD (Figure 2a). The brachydactyly family has a deletion that encompasses portions of EPHA4-TAD as well as the boundary separating it from PAX3-TAD (Figure 2b). The F-syndrome family has inversions or duplication having breakpoints within WNT6/IHH TAD and EPHA4-TAD, encompassing the TAD boundary between these two (Figure 2c). The polysyndactyly family has duplications and deletions within WNT6/IHH TAD, also disturbing its TAD boundary (Figure 2d). All these chromosomal aberrations were re-engineered in a mouse model using the CRSIPR/Cas9 system as well as in hESC (human embryonic stem cells) to map genomic interactions using 4C. The gene expression profile of the locus revealed non-cognate association of the gene promoter with the enhancers. These severe limb malformations clearly resulted from perturbations in the TAD structure and its boundaries, which relocate enhancers with gene promoters. These TAD boundaries are associated with CTCF-loop domains in mouse limbs. This study provides strong evidence that disruption of TADs and TAD boundaries could cause severe developmental disorders in humans. Deciphering the structural basis of X-inactivation in *Caenorhabditis elegans* also revealed the importance of TAD boundaries. A dosage-compensated X-chromosome was observed to have discrete self-interacting 1Mb domains similar to TADs. The boundaries of these TADs are much stronger and are decorated by dosage-compensation condensin complex (DCC) [57]. Loss of DCC complex or deletion of its binding sites results in weaker TAD boundaries and defective dosage compensation. However, these TAD borders were not tested for classical boundary function. These studies indicate that, as in *Drosophila*, TAD borders in other organisms may also harbor insulator function and TAD borders may represent another class of insulator elements. Future studies are expected to delineate their discrete nature from classical insulator elements.

## 4. Stress Response and Insulator-Mediated Chromatin Dynamics

**Figure 3:**
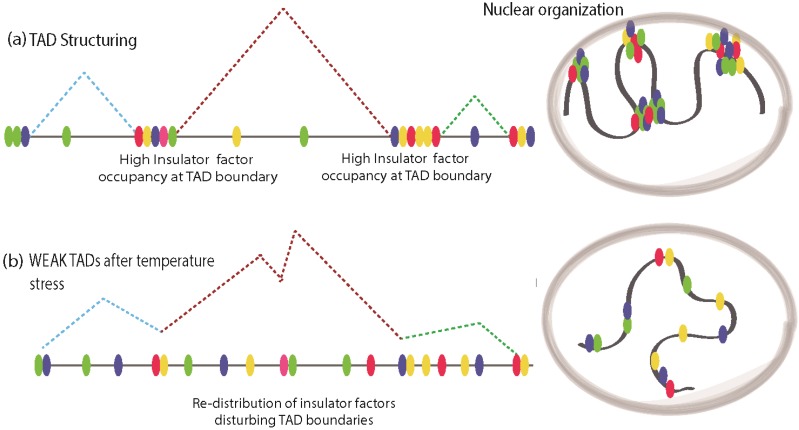
Stress induces redistribution of insulator factors and reconfiguration of chromatin structure. (**a**) Normal TAD is structured by architectural/insulator proteins clustered around their boundaries, maintaining the Megabase scale chromatin structuring, while intra-TAD looping in mediated by discrete motifs for insulator factor binding; (**b**) Under temperature stress, TAD boundaries get weakened due to re-distribution of insulator factors to intra-TAD regions. This may cause promiscuous looping interactions resulting in aberrant transcriptional program.

Cellular stress response is an evolutionarily conserved mechanism that enables organisms to adapt to several environmental challenges such as elevated temperatures, environmental genotoxic agents, and mechanical injury. During conditions of stress, cells undergo a wide range of molecular changes that include the shutting down of many actively transcribed genes and transcriptional activation of several previously silenced genes. Thus cellular response to stress presents a particularly intriguing paradigm to study chromatin dynamics. Recently, insulator factors have been implicated in chromatin re-structuring during temperature stress response in *Drosophila* [58]. It was observed that elevated temperature resulted in disassociation of chromatin-bound CP190, the common co-factor of many *Drosophila* insulator proteins, resulting in global chromatin changes and looping interactions. Indeed, this was shown in a recent report in which heat shock induced re-localization of insulator proteins to ectopic sites, resulting in inter-TAD interactions and large-scale chromatin reconfiguration [59]. Using Hi-C methods, the chromatin dynamics of long-range interactions were analyzed in *Drosophila* Kc167 cells before and after heat shock [59]. Surprisingly, heat shock conditions resulted in more inter-TAD interactions with a concomitant decrease in TAD border strength, whereas before heat shock intra-TAD interactions were predominant. Before heat stress, in Kc167 cells, ChIP-Seq studies using antibodies against a variety of insulator/architectural proteins like BEAF-32, CTCF, Su(Hw), DREF, Rad21 (cohesin), TFIIIC, Cap-H2 (condesin II), Mod(mdg4), CP190, Z4, chromator, L(3)mbt, and Fs1h-L showed that except for Rad21 and Cap-H2, all other architectural/insulator proteins show higher distribution of DNA-bound peaks at TAD boundaries (Figure 3a). Upon heat shock. the distribution of these factors was found to be abundant in interior regions of TAD, at enhancers and promoters away from TAD boundaries, which results in a weakening of TAD boundary strength (Figure 3b). Additionally, the distribution of histone modification marks before and after heat shock in *Drosophila* presented drastic changes in H3K27me3 marks along with ectopic Polycomb (Pc) occupancy at enhancer regions that were previously marked with H3K27ac [59]. This indicates that regulatory regions are repressed upon heat shock and Pc is implicated in the mediated silencing of enhancers upon heat shock. However, it is still not clear if Pc silencing is the direct cause of reorganized insulator factor binding or a secondary response to temperature stress. From these studies, it emerges that chromatin-bound insulators relocate themselves to ectopic sites to re-organize the chromatin upon temperature stress, which leads to changes in transcriptional output. However, in mammals, similar findings are yet to be investigated.

Other studies that have linked insulator factors to stress response include the formation of insulator bodies under conditions of osmotic stress [60]. Insulator bodies are nuclear foci consisting of multiple insulator factors such as Su(Hw), Mod (mdg4), dCTCF, and CP190 that were previously thought to represent functional looping contacts between insulator-bound proteins [61], although this was later refuted in other studies [62]. However, recent studies have indicated that these structures are not formed by chromatin-bound insulator contacts but rather as a result of insulator factors disassociated from chromatin that dramatically alter the chromatin configuration in response to osmotic stress [60]. This osmotic stress response was shown to be specific to insulator factors, as other speckle-forming proteins like Polycomb factors and HP1 foci did not change under osmolarity stress. Finally, it was also shown that the loss of chromatin-bound Su(Hw) protein is reversed once cells were shifted to isotonicity [60]. Although the mechanisms of temperature and osmolarity stress response differ markedly, both of them bring about changes in chromatin dynamics. Taken together, these findings suggest that there is a dramatic disassociation and relocation of chromatin-bound insulator factors during cellular stress. It remains to be seen whether insulator-bound-factors other than Su(Hw) are also relocated in response to osmolarity stress and, like heat stress, if these changes are related to TAD structuring.

## 5. Chromatin Dynamics in Pluripotency and Differentiation

**Figure 4:**
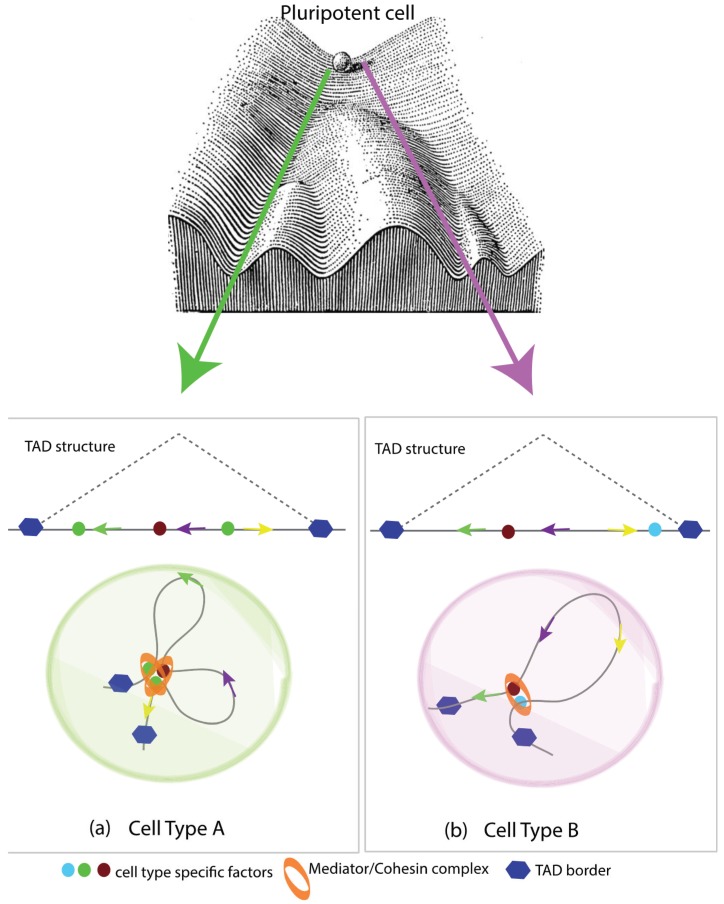
Cell-type-specific genome wiring at the kilobase level. This schematic shows chromatin organization defining the cell type specificity for pluripotent cell along the Waddington’s epigenetic landscape. (**a**) Intra-TAD looping in Cell type A, mediated by the Mediator/Cohesin complex, is instrumental in specifying cell type chromatin folding; (**b**) Despite the overall TAD structure remaining consistent, it is the kilobase-scale interactions in Cell type B that assign the transcriptional program of the cell leading to its lineage specification.

Stem cells have the ability to self-renew and differentiate into multiple lineages of functionally specialized cells in response to appropriate differentiation cues. This cellular plasticity is attributed to several unique structural and functional features of the stem cell genomes. These include hyper-dynamic association of chromatin-interacting factors, disorganized heterochromatin, enhanced global transcriptional activity in coding and non-coding regions, and activation of the second X-chromosome in the female cells [63,64]. The process of differentiation is accompanied by repression in transcription of pluripotency-associated genes, silencing of repeat elements, more stable interaction of chromatin-binding factors, and inactivation of the X-chromosome and formation of distinct heterochromatin foci [64]. Conversely, during reprogramming of differentiated cells into pluripotent state (iPSCs), cells must re-acquire the structural and functional features associated with pluripotency. The mechanism(s) and factors responsible for this genomic plasticity remain elusive. Findings over the past five years have strongly supported a role of insulator factors such as CTCF in shaping the 3D genome architecture in pluripotent cells and its re-organization during differentiation (and *vice versa*). Using ChIA-PET (chromatin immunoprecipitation interaction assay with paired end tagging), Handako *et al*. presented a global and high-resolution CTCF associated interaction map in mouse Embryonic Stem (ES) cells [65]. Their study revealed that CTCF configures the genome in ES cells into distinct chromatin domains and sub-nuclear compartments that bear unique epigenetic features and transcriptional activities. Other studies have shown that CTCF mediated TADs are invariant to stem cells and are conserved not only among diverse cell types but also across species [48]. This indicates that insulator proteins such as CTCF may act as general architectural proteins and that cell-type-specific interactions are mediated by other factors. Indeed, it was recently shown that Cohesin and Mediator but not CTCF play a key role in mediating such cell-type-specific interaction [66]. For example, Cohesin was shown to co-bind genomic sites of CEBPA and estrogen receptor (ER) in HepG2 and MC7 cells respectively, to generate cell specific transcription program [67]. It was also shown that Cohesin is important for mediating chromatin interaction involving ER in MC7 cells. Similarly, Cohesin and Mediator along with the Cohesin loading factor Nipbl (Nipped-B like) were shown to specifically mediate ES cell specific enhancer–promoter interactions [53]. In contrast to CTCF/Cohesin co-bound sites, Cohesin and Mediator co-occupied sites are cell-type-specific and often overlap with pluripotency factors such as Nanog, Oct4, and Sox2 in ES cells. In the same study it was also shown that Mediator and Cohesin occupy different promoters in different cell types and mediate chromatin looping that generates a cell-type-specific expression program (Figure 4a,b).

Since large, megabase-scale TADs are largely conserved between cell types, it is hypothesized that chromatin interactions at sub-megabase level could account for the lineage specific expression programs (Figure 4a,b). To test this, Phillips-Cremins *et al*. used 5C to map interactions in ESCs and Neural progenitor cells (NPCs). They found that large, megabase-scale interactions were enriched in CTCF/Cohesin and were conserved between ESCc and NPCs, a result consistent with previous reports of conservation of TADs across cell types [66]. Cohesin and Mediator co-bound sites were involved in sub-megabase level promoter–enhancer interactions within TADs that were specific to ESCs and NPCs. Depletion of Cohesin or Mediator components resulted in disruption of this cell-type-specific interaction and downregulation of genes within these interactions. It is to be noted that these cell-type-specific interactions are defined by lineage-specific transcription factors during differentiation and are reinforced/maintained by Cohesin/Mediator complex. During T-cell differentiation, multipotent naïve cells, upon receiving antigenic signals, make the lineage choice between Th1 and Th2 cells. These two differential lineages maintain overall similar chromosome interactions, except at the minor looping level, especially at the cytokine locus. Variable genomic contacts between Th1 and Th2 are maintained by DNA-binding, cell-specific transcription factor STAT [68]. In the absence of STAT, the cytokine locus is unable to lose its promiscuous contact with naïve T cells, thus indicating the importance of cell-type-specific factors in chromatin looping rearrangement [68]. Recently, it has been shown that TAD structures are relatively stable across cell types; nevertheless, there are considerable variations accounted for during differentiation [69]. More studies in future are expected to develop a comprehensive and better understanding of what factors and cellular demands lead to TAD re-structuring. 

The reprogramming of somatic cells into pluripotent state is a very inefficient process *in vitro*, and our understanding of the barriers to this process is limited. Only 1%–3% of somatic cells undergo reprogramming to attain pluripotency. Several recent studies have analyzed the interactions in ESCs, iPSCs, and their differentiated progenies and have uncovered chromatin interactions that are unique to successfully reprogrammed cells. One of these studies showed that during reprogramming, overexpressed pluripotency factors (OCT4 and NANOG) are not only bound at endogenous gene loci in successfully reprogrammed cells (iPSCs) but also in un-reprogammed cells [70]. However, specific interaction between pluripotency genes, OCT4, NANOG, and SOX2 was only observed in iPSCs that resulted in transcription from these gene loci [70]. These interactions were shown to be mediated by the Cohesin complex. Using 5C in ESCs, it was shown that Nanog promoter interactions are rearranged upon differentiation and re-established in induced pluripotent cells [71]. A large fraction of Nanog-interacting loci were bound by Mediator or Cohesin and the depletion of either in ECSs resulted in disruption of contacts and re-acquisition of a differentiation-specific interaction pattern. Either loss of chromatin interaction in ESCs or their re-acquisition during induced reprogramming often preceded transcriptional changes, suggesting a functional link between chromatin interactions and transcriptional change. Interaction studies of Oct4 locus using 4C and FISH also suggested a causative role for chromatin interaction and transcriptional activity. Several regions that frequently interacted with the Oct4 locus were identified, which showed that these interactions precede the transcriptional activation of endogenous Oct4 during reprogramming [72]. Klf4 was shown to be critical for these interactions as depletion of Klf4 lead to Cohesin unloading from the Oct4 promoter and loss of transcription of the endogenous Oct4. Studying the long-range interactions in ESC, iPSCs, and differentiated cells revealed that genomic contacts made by pluripotent factors like NANOG are more independent than other classes of contacts such as those mediated by Polycomb proteins. Loss of the Polycomb protein EED resulted in the loss of Polycomb-specific contacts only, preserving the interactions made by pluripotent factors necessary for differentiation [73].

Taken together, the above findings suggest that there are key interactions between pluripotency genes and architectural proteins that are specific to ESCc and iPSCs. During differentiation, looping contacts are specified by cell-type-specific transcription factors within TADs and are mainly maintained by Cohesin/Mediator. However, major loops defining TAD structures coincide with CTCF and Cohesin binding, which are proposed to play a more fundamental role in chromosomal folding.

## 6. Regulation of Insulator Function

Given the idea that insulators compartmentalize the genome into structurally and functionally independent domains of gene activity, it is conceivable that as cells differentiate, insulator-mediated changes in chromatin organization precede or accompany cell differentiation. Such changes may be crucial for the establishment or maintenance of specific patterns of gene expression. If this is true, then cells must possess mechanisms to regulate insulator activity to establish distinct patterns of chromatin organization that are cell type specific. Indeed, several examples exist that demonstrate that insulator function can be modulated at several stages. These mechanisms may involve interfering with protein-protein and/or protein-DNA interactions via protein modification, DNA modification, or competition for DNA binding. Analysis of BEAF-32 binding sites in *Drosophila* provided the first evidence for regulation of insulator activity by competition [74]. Two regions that bind to BEAF-32 act as insulators and are also bound by DREF. BEAF-32 and DREF occupy the sites independently, which leads to a model where these two proteins compete for DNA binding [74]. This observation suggested that the same boundary sequence, when bound to BEAF-32, forms a site of insulation, interfering with enhancer-promoter interaction while DREF binding blocks BEAF-32 and allows enhancer-promoter communication. In a similar manner, covalent modification of the insulator binding sites has been shown to regulate insulator function at the Igf2/H19 locus. DNA methylation of the CTCF binding site on the paternal chromosome blocks its binding, which renders the insulator inactive, resulting in only Igf2 expression in mammals. On the maternal chromosome, the CTCF binding site is not methylated and therefore the insulator is functional, resulting in H19 expression only [75,76]. This mark is imposed during male germ cell development and stably maintained thereafter. This is an example of the epigenetic regulation of insulator activity using a mechanism that involves inhibition of binding of the insulator protein to its DNA sequence.

**Figure 5:**
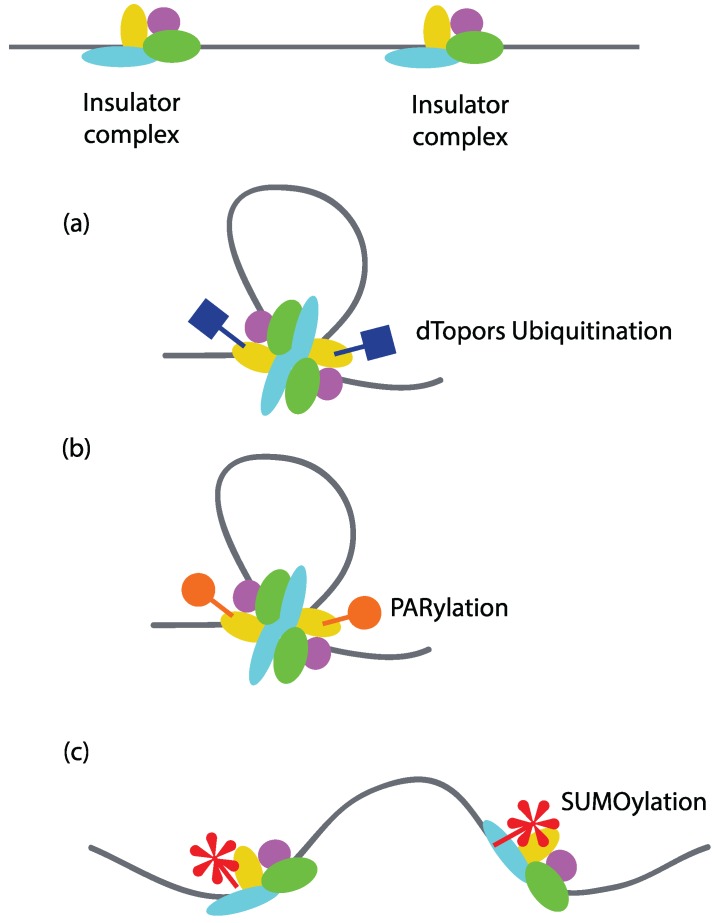
Regulation of insulator activity by covalent modification of insulator factors. Covalent modifications of insulator factors can have both positive and negative effects on insulator-mediated looping interactions. (**a**) Ubiquitination of Su(Hw) by dTopors potentiates its insulator activity by mediating looping interactions; (**b**) PARylation of CTCF regulates its intrachromosomal interactions and hence its insulator activity; (**c**) SUMOylation of Mod(mdg4) and CP190 negatively regulates their activity in *Drosophila* by interfering with the chromatin loop formation.

Covalent modification of insulator proteins may serve as the most predominant means of regulating insulator function. For example, in *Drosophila*, ubiquitination of Su(Hw) by the E3 ubiquitin ligase dTopors increases its insulator activity [77]. A mutation in the dTopors gene reduces the insulator function of Su(Hw), while its over-expression can rescue insulator activity. Since one of the models proposes that insulators function via loop formation by interacting with each other or the components of nuclear structure, it is likely that their function is also regulated through modulation of loop formation (Figure 5a). Poly(ADP-ribosyl)ation or PARylation of CTCF has been shown to regulate CTCF-dependent insulator function in *Drosophila*. Using ChIP-on-chip, it was found that Poly (ADP-ribose) (PAR) co-localizes with around 78% of the CTCF genome-wide binding sites in mouse fetal liver cells. PARylation is necessary for the insulator activity of CTCF at H19-ICR locus, as treatment with 3-aminobenzamide (an inhibitor of PARylation) results in loss of insulator function [78]. Since PARylation has been found to promote protein-protein interactions, it is likely that PARylation regulates insulator activity by promoting protein-protein interaction between insulator sites [79] (Figure 5b). Indeed, PARylation has been shown to be important for the attachment of Nuclear Matrix-associated regions to the nuclear periphery. Inhibition of PARylation results in loss of CP190, Su(Hw), CTCF, and Mod(mdg4) in the nuclear matrix fraction and a two-fold reduction in their binding peaks in ChIP-Seq analysis. Inhibiting the process of PARylation results in loss of intra-chromosomal interactions [80]. When TAD structures were investigated in *Drosophila*, the loss of PARylation did not disrupt the binding of insulator factors on TAD boundaries; however, it is not known if these insulator factors are PARylated while bound to TAD boundaries [80]. 

Modification of Mod(mdg4)2.2 and CP190, the components of the Su(Hw) insulator complex, by SUMO (small ubiquitin-like modification) is another example of regulation of insulator activity by modulating protein–protein interactions [81]. SUMO co-localizes with a number of insulator sites throughout the genome, as shown by immunostaining of polytene chromosomes in *Drosophila*. However, in contrast to PAR, SUMO appears to negatively regulate insulator function as mutations in the SUMOylation pathway result in enhanced insulator activity (Figure 5c). Furthermore, over-expression of SUMOylation pathway components has been shown to disrupt the formation of insulator bodies in diploid nuclei from third instar larvae of *Drosophila*. This observation suggests that SUMOylation regulates insulator function by interfering with the ability of insulators from different sites to interact with each other (Figure 5c).

A few years ago, it was shown that RNAi machinery also plays a role in regulating interaction between insulator-associated proteins. The Su(Hw) insulator requires RNAi machinery to make RNAs that mediate the interactions between individual insulator sites to form insulator bodies. Mutations that affect the components of RNAi machinery and presumably impair the production of these RNAs affect insulator function. For example, loss of function and over-expression studies showed that the helicase Rm62 has a negative effect on insulator function, whereas Argonaute (Ago) proteins facilitate their activity [82]. Moshkovich and colleagues showed an RNAi-independent role of Argonaute-2 in dCTCF/CP190-dependent insulator function [83]. Ago2 was shown to physically interact with dCTCF and CP190 and mutation in Ago2 results in a reduction of chromosomal looping interaction and loss of insulator activity.

Apart from the aforementioned ways of regulating insulator activity, there are other modes through which insulator function can be modulated. For example, the *Abdominal-B (Abd-B)* locus in *Drosophila* consists of a series of developmentally regulated enhancers, silencers, and insulators. The question of how a correct enhancer targets the *Abd-B* gene in a given cell type is intriguing. Several insulator elements in the *Abd-B* region have been shown to exhibit insulator bypass (pairing of insulators), resulting in loss of insulator function [84,85,86,87]. Utilizing this mechanism, specific enhancers can be targeted to their cognate promoters in a particular cell type and at the correct developmental stage. Another way to achieve developmentally regulated expression of the *Abd-B* locus is through Promoter Targeting Sequences (PTS). Such sequences have been found at the 3' region of two of the insulator elements, Fab-7 and Fab-8, and allow enhancers to overcome dCTCF and Su(Hw) enhancer-blocking activity in transgenic assays [88,89]. Nearby sequences affecting insulator function have also been found in vertebrates. Thyroid response elements (TRE) found upstream of the chicken *lysozyme* gene and TRE upstream of the *c-myc* gene have been found to reduce CTCF enhancer-blocking activity [90]. CTCF remains bound to DNA in the presence of the hormone, indicating that TRE must inactivate CTCF. An additional layer of regulation of insulation comes from various interacting partners [91]. CHD8 enhances insulator activity, whereas Kaiso has a negative effect on the CTCF-mediated enhancer blocking [92,93].

Given the vast number of strategies for regulating insulator function both at the level of DNA binding and in modulating protein–protein interactions that mediate loop formation, insulator activity is dynamic. Interfering at the level of DNA binding could be a more permanent form of regulation, whereas protein competition and modification are reversible modes of regulation that could be adapted by cells to respond to changing needs during development and differentiation or during cellular stress.

## 7. Conclusions and Future Directions

Recent studies have extensively mapped chromatin interactions at multiple spatial scales and greatly expanded our understanding of the organizational role of insulators/architectural proteins in 3D genome organization. Several questions remain unanswered that need to be addressed in future studies. For example, it remains to be established whether gene expression is a cause or effect of such interactions, although recent studies have provided some evidence in favor of the latter [72]. Moreover, chromatin interactions have been predominantly analyzed in cases of gene activation; equally important will be to map looping interactions that result in gene silencing, which may be important not only for maintaining pluripotency (by silencing differentiation genes) but also for generating/maintaining cell type specificity. It is also important to keep in mind that interactions maps are generated from a large pool of fixed cells and therefore do not provide the dynamics of those interactions. Future studies aiming to image the dynamic clustering of insulator factors might throw more light on this proposition. Perhaps the most important challenge in the field will be to link chromatin interactions to their functional outcomes. Recent advancements in gene editing technologies such as the CRISPR-Cas9 system will allow for perturbing or manipulating these interactions to understand their underlying functions. Finally, the perturbation of TADs and TAD boundaries as the underlying cause of human genetic defects is a powerful example of how chromatin organization is relevant to human disease. Therefore, it is essential to consider the impact of genetic defects on chromatin topology in order to fully understand the molecular mechanism of their pathogenesis.
